# The Role of Exposure to Phthalates from Polyvinyl Chloride Products in the Development of Asthma and Allergies: A Systematic Review and Meta-analysis

**DOI:** 10.1289/ehp.10846

**Published:** 2008-03-31

**Authors:** Jouni J.K. Jaakkola, Trudy L. Knight

**Affiliations:** Institute of Occupational and Environmental Medicine, University of Birmingham, Birmingham, United Kingdom

**Keywords:** allergy, asthma, phthalates, polyvinyl chloride

## Abstract

**Background:**

Phthalates from polyvinyl chloride (PVC) plastics may have adverse effects on airways and immunologic systems, but the evidence has not been reviewed systematically.

**Objective:**

We reviewed the evidence for the role of exposure to phthalates from PVC products in the development of asthma and allergies.

**Methods:**

We conducted a Medline database search (1950 through May 2007) for relevant studies on the respiratory and allergic effects of exposure to phthalates from PVC products.

**Results:**

We based this review on 27 human and 14 laboratory toxicology studies. Two mouse inhalation experiments indicated that mono-2-ethylhexyl phthalate (MEHP) has the ability to modulate the immune response to exposure to a coallergen. The data suggested a no observed effect level of 30 μg MEHP/m^3^, calculated to be below the estimated level of human exposure in common environments. Case reports and series (*n* = 9) identified and verified cases of asthma that were very likely caused by fumes emitted from PVC film. Epidemiologic studies in adults (*n* = 10), mostly small studies in occupational settings, showed associations between heated PVC fumes and asthma and respiratory symptoms; studies in children (*n* = 5) showed an association between PVC surface materials in the home and the risk of asthma [fixed-effects model: summary odds ratio (OR), 1.55; 95% confidence interval (CI), 1.18–2.05; four studies] and allergies (OR, 1.32; 95% CI, 1.09–1.60; three studies).

**Conclusions:**

High levels of phthalates from PVC products can modulate the murine immune response to a coallergen. Heated PVC fumes possibly contribute to development of asthma in adults. Epidemiologic studies in children show associations between indicators of phthalate exposure in the home and risk of asthma and allergies. The lack of objective exposure information limits the epidemiologic data.

Polyvinyl chloride (PVC) plastics are used extensively for a very wide range of purposes, such as interior surfaces, food wrappers, and covering of crops in agriculture. Extensive use of PVC is related to its stability and flexibility, which is achieved by incorporation of plasticizers. More than 300 different types of plasticizers have been identified, and between 50 and 100 are used commercially. Phthalates, diesters of benzenedicarboxylic acid (phthalic acids), constitute the most commonly used plasticizers. In Western Europe, about 1 million tons of phthalates are produced each year, of which approximately 900,000 tons are used to plasticize PVC (Plasticisers Information Center 2007). The most common are diisononyl phthalate (DiNP), diisodecyl phthalate (DiDP), and di-2-ethyl-hexyl phthalate (DEHP).

Phthalate compounds leach, migrate, or gas out from PVC-containing items into air, dust, water, soils, sediments, and food and have become ubiquitous environmental contaminants (Clark et al. 2003). Recent studies of human urine samples in industrialized countries have highlighted the large extent of population exposure to various phthalates (Wormuth et al. 2006). Diet, particularly fatty food (e.g., dairy, fish, oils), is the main source of DEHP exposure in the general public (Clark et al. 2003; Meek and Chan 1994; Peterson and Breindahl 2000; Wormuth et al. 2006); other sources include consumer products and medical procedures. Although phthalates have low volatility, they off-gas and are present in residential indoor air and dust (Adibi et al. 2003; Rudel et al. 2003). Dampness has been shown to enhance degradation of PVC flooring, resulting in elevated indoor air concentrations of 2-ethyl-1-hexanol, a hydrolysis product of DEHP (Norbäck et al. 2000; Tuomainen et al. 2004). Thus, although most exposure to phthalates has traditionally been thought to be ingestion, other routes—such as dermal, parenteral, and, in particular, inhalation—may be important. However, the proportional contribution from various sources and routes of exposure is unknown.

Evidence has accumulated for association of harmful health effects with exposure to phthalates, particularly DEHP (Hauser and Calafat 2005), raising public concerns and debates. Although emphasis has been given to potential adverse reproductive and carcinogenic effects, phthalates may have adverse effects on airways and immunologic systems, but the evidence has not been reviewed systematically. The objective of this study was to review the evidence for the role of exposure to phthalates from PVC products in the development of asthma and allergies and to make recommendations for future research.

## Methods

### Search strategy and inclusion criteria

We performed a systematic literature search of the Medline database (National Library of Medicine, Bethesda, MD, USA) from 1950 through May 2007 using the search command “[phthalates OR polyvinyl chloride] AND [asthma OR allergy].” We identified and screened a total of 54 references in two phases: initially from abstracts, to eliminate the obviously irrelevant ones, and then from full publications of the remaining references. To be included in the review, the study had to focus on the effects of phthalate exposure on the human respiratory system, or on an immunologic parameter in animal or *in vitro* tests.

### Data extraction

We divided the relevant publications into three categories: mechanistic toxicology studies, case reports and case series on occupational asthma, and epidemiologic studies and human controlled experiments. From the epidemiologic study reports, we recorded the most relevant characteristics of each selected publication and considered the possibility for its inclusion in the meta-analysis.

### Statistical methods

In the meta-analysis, we calculated summary effect estimates by using fixed- and random-effects models. The fixed-effects model applied the Mantel-Haenszel method (Mantel and Haenszel 1959) with inverse variances of individual effect estimates as weights. The random-effects model applied the method of DerSimonian and Laird (1986). We calculated the natural log and its SE for the effect estimates from the raw data or from the confidence intervals (CIs) presented in the articles. When available, we preferred the adjusted effect estimates over the crude estimates. We used the “meta” command (Sterne et al. 2001) to run the fixed- and random-effects models on Stata 8.2 (StataCorp LP, College Station, TX, USA). We tested heterogeneity between study-specific effect estimates using *Q* statistics and chi-square distribution (Sterne et al. 2001). The two meta-analyses provided homogeneous results; therefore, we did not need to elaborate determinants of heterogeneity.

## Results

Of the 54 publications we identified in the Medline search, we eliminated 27 as obviously irrelevant. We retrieved copies of the remaining 27 publications and reviewed them in detail. We included an additional 14 references cited in these articles that fulfilled our inclusion criteria. We based the systematic review on evidence from 41 publications, which we categorized into mechanistic toxicology studies (*n* = 14), case reports and case series on occupational asthma (*n* = 9), epidemiologic studies (*n* = 17), and a human controlled experiment (*n* = 1).

### Mechanistic toxicology studies

In the Medline search, we identified eight publications categorized as mechanistic toxicology (Butala et al. 2004; Doelman et al. 1990; Glue et al. 2005; Hansen et al. 2007; Jepsen et al. 2004; Larsen et al. 2001a, 2002a, 2003), and their reference lists yielded six additional articles (Bally et al. 1980; Glue et al. 2002; Klimisch et al. 1992; Larsen et al. 2001b, 2004; Nakamura et al. 2002). PubMed and Toxline (National Library of Medicine) searches for mechanistic studies identified no additional studies articles.

**Figure 1:**
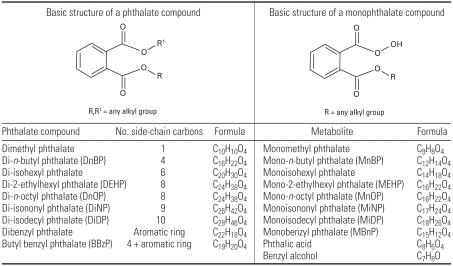
Chemical formulas of phthalates.

Five articles described mechanistic studies of a mouse model used to investigate whether coadministration of a phthalate compound modulates the immune response to an allergen (ovalbumin) (Hansen et al. 2007; Larsen et al. 2001a, 2001b, 2002b, 2003). In these studies commercial plasticizers [diphthalates and butyl benzyl phthalate (BBzP)] and their main metabolites (monophthalates, phthalic acid, and benzyl alcohol) were used as test compounds. Formulas for common phthalates and their metabolites are shown in Figure 1 [an illustration of a metabolic scheme is presented in Appendix Figure A, available online in Supplemental Material (http://www.ehponline.org/members/2008/10846/suppl.pdf); scheme adapted from Fredericksen et al. 2007].

The initial studies (Larsen et al. 2001a, 2001b, 2002, 2003) were designed to investigate whether phthalates have adjuvant or immunosuppressive properties without considering true human exposure routes or concentrations. Compounds were administered by subcutaneous injection: either a 100-μL dose of a solution containing 1 μg ovalbumin and 0, 1, 10, 100, or 1,000 μg phthalate/mL (Larsen et al. 2001a) or a 50-μL dose of a solution of 1 μg ovalbumin and 0, 2, 20, 200, or 2,000 μg phthalate/mL (Larsen et al. 2001b, 2002, 2003). Responses were assessed by enzyme-linked immunosorbant assay of IgE, IgG1, and IgG2a antibodies. Stimulation of the T_H_2 pathway, which is predominant in type I allergies, involves increased production of IgE and IgG1 antibodies, whereas stimulation of the T_H_1 response, predominant in type IV allergies, involves IgG2a antibodies (Larsen et al. 2001a). A statistically significant increase or decrease in antibodies, by phthalate, was defined as an adjuvant or an immunosuppressive effect, respectively.

Individual phthalates varied in the antibody class they induced and in their potency of adjuvancy. The concentrations of phthalates that provoked a statistical increase in antibodies in response to one booster of ovalbumin were the monophthalates mono-2-ethylhexyl phthalate (MEHP), 10 μg/mL (IgE); mono-*n*-octyl phthalate (MnOP), 100 μg/mL (IgE) and 10 μg/mL (IgG); monoisononyl phthalate (MiNP), 100 μg/mL (IgE) (Larsen et al. 2001a); and the diphthalates DEHP, 2,000 μg/mL (IgG1) (Larsen et al. 2001b); di-*n*-butyl phthalate (DnBP), 200 μg/mL (IgG1); DiNP, 200 μg/mL (IgE) and 200 μg/mL (IgG1); di-*n*-octyl phthalate (DnOP), 2,000 μg/mL (IgG1) and 2,000 μg/mL (IgE) (compared with the corresponding control group; Larsen et al. 2002); and DiDP, 2,000 μg/mL (IgE) (compared with only the cumulated ovalbumin control groups; Larsen et al. 2002). Mono-*n*-butyl phthalate (MnBP), monobenzyl phthalate (MBnP) (Larsen et al. 2001a), and BBzP (Larsen et al. 2003) produced no adjuvancy. IgG2 antibodies were not induced by any of the phthalates tested.

The extent of adjuvancy provoked by individual phthalates was thus shown to be structure related (Jepsen et al. 2004; Larsen et al. 2001a). In mice, monophthalates with 8 (MEHP, MnOP) or with 9 (MiNP) side-chain carbons caused a greater increase in antibodies than those with 4 (MnBP), 7 (MBnP), or 10 [monoisodecyl phthalate (MiDP)] side-chain carbons (Larsen et al. 2001a). *In vitro* studies in a human epithelial cell line (Jepsen et al. 2004) comparing the cytokine stimulation potencies of monophthalates showed MEHP, MnOP, and MiNP to be more potent inducers of cytokines than MnBP or MBnP (Jepsen et al. 2004); in contrast to the *in vivo* work, MiDP was a potent inducer of cytokines.

The degree of increase (and sometimes the class) of antibodies was generally shown to be concentration dependent. For example, DnOP produced a concentration-dependent increase in production of IgG1 but not IgE (Larsen et al. 2002). Exposure of mice to 200 μg/mL DiNP following a single booster of ovalbumin produced non–concentration-dependent increased levels of IgG1 and IgE; however, after two boosters, a concentration-dependent adjuvancy in IgG1, but no adjuvancy in IgE, was observed (Larsen et al. 2002). Furthermore, increasing the concentration of some phthalates (DnBP and DiNP, from 200 to 2,000 μg/mL) caused a decrease in antibody production (Larsen et al. 2002). Exposure of mice to benzyl alcohol, a metabolite of BBzP, also caused a significant reduction in IgG1 release compared with that in control mice given ovalbumin only (Larsen et al. 2003).

Subsequent work in mice used the inhalation route of administration to mimic human exposure to airborne phthalates (Hansen et al. 2007; Larsen et al. 2004). Mice received long-term exposures (20 min for 5 days/week for 2 weeks, then once weekly for 12 weeks) to aerosols of ovalbumin with 0.03 or 0.4 mg MEHP/m^3^ (Hansen et al. 2007). Although no effects were observed on lung function parameters or in the levels of IgE or IgG2, the levels of serum IgG1 and the number of lymphocytes and eosinophils in bronchoalveolar lavage (BAL) fluid were significantly increased by exposure to 0.4 mg MEHP/m^3^, relative to those in the ovalbumin-only group (Hansen et al. 2007). No modulation of immune response was apparent after exposure to 0.03 mg MEHP/m^3^, and the group established this as the no observed effect level (NOEL) (Hansen et al. 2007) to assess the relevance of their findings to normal exposures of humans. They used the NOEL of MEHP (0.03 mg/m^3^) to estimate the parent compound (DEHP) equivalence (3 mg DEHP/m^3^); using median indoor air and worst-case exposures of 0.04 μg and 1.2 μg DEHP/m^3^, they estimated a margin of exposure (i.e., the ratio of no effect concentration DEHP/human exposure concentration DEHP) to be between 2,500 and 75,000 (Hansen et al. 2007).

Larsen et al. (2004) measured indicators of modulation of immune response to ovalbumin by MEHP after short-term exposures (60 min; 0.3–43.6 mg/m^3^ dose). They reported a concentration-dependent decrease in tidal volume (NOEL ~ 300 μg/m^3^) and an increased number of alveolar macrophages, but no change in numbers of neutrophils, lymphocytes, eosinophils, or epithelial cells in BAL fluid. By calculating a worst-case human exposure value to DEHP (300 μg/m^3^) (using on-floor and airborne dust levels in Oslo and Denmark and personal air samples from Poland) and extrapolation of ventilation rate and lung surface area in mice to human parameters, this group concluded that no airway irritation can be expected from indoor exposure to DEHP in nonoccupational settings.

Phthalate compounds also showed an immunosuppressive effect on the immune response to ovalbumin in *in vivo* and *in vitro* studies (Jepsen et al. 2004; Larsen et al. 2001a). NOEL values determined for these effects (Jepsen et al. 2004; Larsen et al. 2001a) showed structure-related variation between individual compounds (Larsen et al. 2001a). In general, the longer the carbon chain, the lower the NOEL. The NOELs for suppression of IgE and IgG1 for individual monophthalates were 100 μg/mL (MEHP), 100 μg/mL (MnOP), 10 μg/mL (MiDP), and 100 μg/mL (MiNP, IgE) and 10 μg/mL (MiNP, IgG1) (Larsen et al. 2001a). MnBP and MBnP produced no immunosuppression (Larsen et al. 2001a). Similarly, *in vitro* studies using human cell lines showed that MiDP, MiNP, MEHP, and MnOP were more suppressive of interleukin (Il)-6 and Il-8 than were MBnP and MnBP (Jepsen et al. 2004) and that, in most cases, the NOEL for suppression was the same as the concentration that induced maximum cytokine production.

For this group of articles (Jepsen et al. 2004; Larsen et al. 2002, 2003), laboratory experiments in mice and human lung epithelial cells showed a modulatory effect by phthalate compounds on the immune response to a coallergen. In general, lower concentrations of phthalates showed adjuvancy, and higher concentrations showed immunosuppression (Jepsen et al. 2004; Larsen et al. 2002, 2003).

The remaining mechanistic toxicologic studies were of various cellular and physiologic aspects of immune response induced by phthalates. In mice, Butala et al. (2004) used topical administration of diphthalates [DEHP (25%, 50%, and 100%), DiNP, BBzP, and di-isohexyl phthalate (100%)], without coallergen, to investigate their effects on levels of serum IgE and lymph node cytokines (Il-4 and Il-13), as an indication of their respiratory sensitizing potential. The diphthalates produced no significant increases in IgE or cytokines, and Butala et al. (2004) concluded that diphthalates have little, if any, respiratory sensitizing potential.

*In vitro* 24-hr incubation of a human monocytic cell line (THP-1) with a dilution series (0.2, 2.0, 20, and 200 μg/mL) of MEHP, MnBP, or MBnP, or 48-hr incubation of human peripheral blood mononuclear cells with 220 μg/mL MnOP, MiNP, or MiDP produced no increased cytokine responses (Glue at al. 2002). Bally et al. (1980) reported an increase in phagocytosis and release of lysosomal enzymes in rabbit alveolar macrophages treated with DEHP in concentrations comparable with those found in stored blood. In rat basophilic leukemia cells (RBL-2H3 mast cells), antigen-induced degranulation (β-hexosaminidase release) increased by raised cytosolic calcium ion concentration induced by coincubation with phthalates (DnBP to a greater extent than diisobutyl phthalate or DEHP) (Nakamura et al. 2002). Glue et al. (2005) reported rapid histamine release after incubation of human peripheral blood mononuclear cells (containing 0.1–1% basophils) with DEHP or MEHP, and anti-IgE antibody costimulant. The 8-carbon phthalates (DEHP, MEHP, MnOP, and DnOP) were the strongest histamine release potentiators, whereas 4-, 9-, or 10-carbon phthalates (MnBP, DnBP, MiNP, DiNP, MiDP, and DiDP) caused no or low induction of histamine (Glue et al. 2005). Alternative costimulants [formyl-methionyl-leucyl-phenylalanine, a bacteria-derived peptide; calcium ionophore; and cat hair extract) also provoked increased release of histamine compared with controls (Glue et al. 2005). In rats, DEHP inhalation (estimated doses of 230, 11, or 2.3 mg/kg/day for males and 360, 18, or 3.6 mg/kg/day for females) resulted in a significant increase in relative lung weights and increased foam cell proliferation and thickening of the alveolar septi (Klimisch et al. 1992). MEHP (0.1 mmol/L), but not DEHP or phthalic acid (concentrations up to 1 mmol/L), induced hypersensitivity to methacholine-induced contraction of rat tracheal muscle (Doelman et al. 1990), an effect that the authors suggested may result in clinical bronchial hyperreactivity, characteristic of asthma, if it occurred in humans.

In summary, animal studies show the potential of phthalate compounds to cause modulation of the immune response, and indicate the mechanism involved (Hansen et al. 2007; Larsen et al. 2001a, 2001b, 2002b, 2003b, 2004). The NOEL suggested from these studies (0.03 mg/m^3^ MEHP) is estimated to be substantially higher than normal human exposures (Hansen et al. 2007; Larsen et al. 2004). Uncertainty remains, however, regarding the potential human exposure to PVC degradation products in occupational settings. Published extrapolations of experimental doses to human exposures suggest that *in vivo* findings in mice have depended upon higher exposures than those encountered by humans in the environment (Hansen et al. 2007; Larsen et al. 2004).

### Case reports and case series

The primary search identified eight articles on case reports or case series related to PVC exposure (Andrasch and Bardana 1976; Brunetti and Moscato 1984; Butler et al. 1981; Cipolla et al. 1999; Lee et al. 1989; Moisan 1991; Muñoz et al. 2003; Pauli et al. 1980), and the references provided one more article (Sokol et al. 1973). The 29 cases described in these nine articles are summarized in Appendix Table A, available online in Supplemental Material (http://www.ehponline.org/members/2008/10846/suppl.pdf).

Four case reports (Andrasch and Bardana 1976; Muñoz et al. 2003; Pauli et al. 1980; Sokol et al. 1973) identified and verified cases of asthma that are very likely caused by fumes from hot-wire cutting of PVC film or in combination with fumes from thermoactivated price labels. Strong work-related respiratory symptoms have been shown to occur without specific airway reactivity to these fumes (Butler et al. 1981).

Other cases of occupational asthma have been linked to exposure to a heated mixture of PVC and DEHP in production of artificial leather (Brunetti and Moscato 1984); fumes from residential fire involving plastic laminates, refrigerator components, wall coverings, and synthetic drapery material (Moisan 1991); unheated PVC resin mixtures (Lee et al. 1989); and dioctyl phthalate from work with a conveyor belt for bottle stoppers (Cipolla et al. 1999).

### Epidemiologic studies

We identified 12 articles on relevant epidemiologic studies, and an additional 5 from their reference lists, totaling 17 articles (Tables 1 and 2): 10 articles on adults and 7 on children.

#### Adult studies

All 10 studies in adults assessed the relationship between PVC-related occupational exposure (meat wrappers, hospital and office workers, firefighters, PVC processors) and the risk of asthma, allergies, or related respiratory effects (Andrasch et al. 1976; Brooks and Vandervort 1977; Eisen et al. 1985; Falk and Portnoy 1976; Jaakkola et al. 2006; Markowitz 1989; Nielsen et al. 1989; Norbäck et al. 2000; Polakoff et al. 1975; Tuomainen et al. 2004), and one of the studies (Jaakkola et al. 2006) also examined the role of home exposures. Of these studies, 6 were cross-sectional, and 4 were longitudinal; 1 was a repeated cross-sectional study, 2 were cohort studies, and 1 was a population-based incident case–control study.

Four of the cross-sectional studies (Andrasch et al. 1976; Brooks and Vandervort 1977; Falk and Portnoy 1976; Polakoff et al. 1975) and one cohort study (Eisen et al. 1985) assessed the relationship between PVC-fume exposure in individuals employed as meat wrappers and respiratory symptoms and illnesses. Polakoff et al. (1975) published the first epidemiologic study on respiratory effects related to meat wrapping. In that study, 17 meat wrappers exposed to pyrolysis products of PVC had a higher prevalence of cough, phlegm, hay fever, and asthma than did the reference group of 21 subjects (Table 1). The exposed group also demonstrated relative decreases in forced expiratory volume in 1 sec (FEV_1_) and forced expiratory flow 50% (FEF_50_) after one shift of work. Falk and Portnoy (1976) conducted a cross-sectional study of 445 Houston supermarket workers, comparing two reference groups (150 checkers and 150 meat cutters) with 145 meat wrappers exposed to thermal decomposition fumes of PVC film wrap. They reported an increased prevalence of respiratory symptoms, including shortness of breath, wheezing, and chest pain, among the meat wrappers, as well as increased occurrence of upper respiratory symptoms (dry or sore throat, stuffy or runny nose, coughing, chest tightness) and eye symptoms (burning, itchy, or tearing eyes) and acute respiratory tract illness (pleurisy, bronchitis, and pneumonia) (Table 1). In a study of 96 meat wrappers (Andrasch et al. 1976), 69% were reported to present work-related effects of the respiratory tract, mucous membranes, and systemic symptoms, with prevalences of 57%, 61%, and 16%, respectively. In bronchial provocation tests of symptomatic workers to PVC fumes, 3 of 11 developed a mean decline of 25% in FEV_1_. Exposure to adhesive fumes from price labels resulted in a mean decline of 49% in FEV_1_ and 40% in forced vital capacity (FVC) of prechallenge values in 9 of 13 workers. In a study of 24 meat wrappers and 20 office clerks, Brooks and Vandervort (1977) reported an increased prevalence of asthma/allergy and upper and lower respiratory tract symptoms among exposed meat wrappers, but no differences were found between preshift and post-shift lung functions. Eisen et al. (1985) assessed pulmonary function before, during, and after shift in 83 workers in the retail food industry; 40 workers using hot-wire wrapping and exposed to PVC emissions were compared with 43 workers not using hot-wire wrapping. They found no association between acute FEV_1_ change and hot-wire wrapping, but the interaction term for hot-wire exposure and asthma/allergy was borderline significant, suggesting that workers with asthma or allergy may constitute a susceptible group.

Of these epidemiologic studies, all provide evidence for a high prevalence of work-related eye and upper and lower respiratory tract symptoms among meat wrappers. Those studies conducted with a suitable reference group show that the risk is substantially higher among the exposed (Table 1). The findings on the effects on lung function were inconsistent; Polakoff et al. (1975) found an increased preshift–postshift decline in FEV_1_ and FEF_50_, whereas Brooks and Vandervort (1977) found no changes in any of the studied lung function parameters. Eisen et al. (1985) found no general longitudinal decline over time but provided evidence of such an effect among workers with asthma or allergy.

Two additional epidemiologic studies provide evidence that pyrolysis products of PVC may increase bronchial reactivity and asthma symptoms. In a cohort study, Markowitz (1989) reported that 66 firefighters exposed to burning PVC at a warehouse fire had an increased risk of asthma-related respiratory symptoms 5–6 weeks and 22 months after the exposure. In a study of 39 PVC-processing plant workers, Nielsen et al. (1989) found that machine attendants exposed to PVC thermal degradation products and phthalic acid esters had a higher prevalence of asthma, rhinitis, and eye and respiratory symptoms than did an internal, unexposed reference group. One of 20 exposed workers had a positive specific bronchial provocation test, and 1 had specific IgG against phthalic anhydride.

Three epidemiologic studies assessed the potential role of PVC surface materials and the development of asthma, allergies, and related health problems (Jaakkola et al. 2006; Norbäck et al. 2000; Tuomainen et al. 2004). Norbäck et al. (2000) studied 87 workers in four Swedish geriatric hospitals. Two hospitals had signs of dampness-related degradation of DEHP in PVC flooring, indicated by visual observations and indoor air measurement of 2-ethyl-1-hexanol, a hydrolysis product of DEHP. The risk of asthma symptoms was greater among the 50 exposed workers than among the 37 workers in the two reference hospitals, with an adjusted odds ratio (OR) of 8.6 (95% CI, 1.3–56.7). Tuomainen et al. (2004) identified an office building with severe dampness problems and damaged PVC flooring that had indoor air 2-ethyl-1-hexanol concentrations of 1–3 μg/m^3^. They conducted a repeated cross-sectional study of the workforce before and 4 years after the intervention, which included removal of floor coverings, adhesive, and smoothing layers. The prevalence of respiratory, conjunctival, and nasal symptoms was reduced after the intervention. Eight new cases of asthma occurred during a 4-year period, which is 9.2 times more than expected. In another study, Tuomainen et al. (2006) conducted controlled challenge tests using degraded PVC products in 10 workers who had previously experienced respiratory symptoms related to this office building, 5 of which had doctor-diagnosed asthma (Tuomainen et al. 2006). After the challenge, they found a 50% increase in the number of symptoms reported compared with 0% before the challenge (*p* < 0.029); the challenge did not influence the lung function parameters, exhaled nitrous oxide (NO), nasal NO, or NO in nasal lavage. The exhaled samples contained 2-ethyl-1-hexanol. In a population-based incident case–control study of 521 new asthma cases and 932 population controls (Jaakkola et al. 2006), the risk of asthma was related to the presence of plastic wall materials at work (< 50% wall surface: adjusted OR, 1.26; 95% CI, 0.49–3.22; ≥ 50% wall surface: adjusted OR, 2.43; 95% CI, 1.03–5.75) compared with no plastic materials.

#### Studies in children

The systematic search identified seven articles from five epidemiologic studies conducted in Norway (Jaakkola et al. 1999; Øie et al. 1999), Finland (Jaakkola et al. 2000), Sweden (Bornehag et al. 2004a, 2004b, 2005), and Russia (Jaakkola et al. 2004), with each article providing effect estimates on the relationship between residential exposure to PVC-related emissions and the risk of asthma, allergies, or related respiratory effects (Table 2). The Finnish (Jaakkola et al. 2000) and the Russian studies (Jaakkola et al. 2004) and the first phase of the Swedish study (Bornehag et al. 2004a, 2005) were population-based cross-sectional studies. The Norwegian study (Jaakkola et al. 1999; Øie et al. 1999) was a one-to-one matched case–control study, and the second phase of the Swedish study (Bornehag et al. 2004b) was a case–control study. The cross-sectional studies based both exposure and outcome assessment on parent-administered questionnaire information. Both case–control studies (Bornehag et al. 2004b; Jaakkola et al. 1999) used trained investigators to assess the sources of exposure and other residential factors; in addition, the Swedish study (Bornehag et al. 2004b) measured content of six phthalates in the dust samples taken from the child’s bedroom. In the Norwegian study, Jaakkola et al. (1999) based the assessment of bronchial obstruction on information from the project pediatrician, family physician records, follow-up questionnaires, and medical records. They defined bronchial obstruction as two or more episodes of symptoms and signs of bronchial obstruction or one episode lasting > 1 month. A committee of three senior pediatricians made the final decision. In the Swedish case–control study (Bornehag et al. 2004b), the cases were defined as individuals reporting two of three conditions during the past 12 months, including at least two incidents of eczema, or wheezing, or rhinitis without a cold and, in the 1.5-year follow-up, at least two of three possible symptoms or conditions. The cases and controls underwent a medical examination.

In the Norwegian matched case–control study of 251 cases of bronchial obstruction and 251 controls (Jaakkola et al. 1999), the risk of bronchial obstruction was greater in the presence of PVC in the floors (adjusted OR, 1.89; 95% CI, 1.14–3.14). The risk of bronchial obstruction was also related to a plasticizer exposure index (adjusted OR, 2.72; 95% CI, 1.50–4.91). Further analyses showed that the relationship of bronchial obstruction to a plasticizer exposure index was stronger in homes with low air change than in those with high air change (Øie et al. 1999).

In the Finnish population-based cross-sectional study of 2,568 children 1–7 years of age, the risks of wheezing, persistent phlegm, weekly nasal congestion or excretion, and respiratory infections were related to the presence of plastic wall materials at home (Jaakkola et al. 2000).

In the Swedish cross-sectional study of 10,851 children (Bornehag et al. 2004b), the risks of asthma, rhinitis, wheezing, or cough past 12 months of age were not related to the presence of PVC flooring when adjusting for personal, family, and housing characteristics (ORs, 0.90–1.15). Water leakage and other indicators of dampness and mold problems were independent determinants of asthma and rhinitis. However, the combination of water leakage and the presence of PVC flooring was the strongest determinant of doctor-diagnosed asthma (adjusted OR, 1.48; 95% CI, 1.11–1.98) and rhinitis (adjusted OR, 1.38; 95% CI, 1.15–1.65), compared with no leakage and PVC flooring. Bornehag et al. (2004b) conducted a case–control study on a population recruited from a 1.5-year follow-up of the cross-sectional study. The 198 cases included subjects with persistent allergic symptoms present both at baseline and on follow-up surveys (106 with asthma, 79 with rhinitis, and 115 with eczema), and 202 controls were free of these symptoms. The cases and controls underwent a medical examination, and a trained technician assessed the characteristics of their homes. The case status was related to the presence of PVC flooring in the bedroom (adjusted OR, 1.59; 95% CI, 1.05–2.41). The dust concentrations (milligrams per gram dust) of six phthalates—diethyl phthalate, di-isobutyl phthalate, DnBP, BBzP, DEHP, and DiNP—were determined. Median house dust concentrations of BBzP were higher in the bedrooms of cases (0.899 mg/g dust) than in those of controls (0.723 mg/g dust). The risk of allergic rhinitis and eczema was related to the house-dust BBzP concentrations, whereas the risk of asthma was related to the concentration of DEHP (Bornehag et al. 2004b).

Four studies provided comparable estimates of the relation between presence of PVC surface materials and the risk of asthma, and three studies focused on allergic rhinitis or allergies. This enabled two formal meta-analyses. In the fixed-effects model, the summary OR of asthma was 1.55 (95% CI, 1.18–2.05), and the study-specific effect estimates were homogeneous [*Q* statistic (3 degrees of freedom), 1.701; *p* = 0.637]. The summary OR of allergic rhinitis was 1.32 (95% CI, 1.09–1.60), again with homogeneous study-specific effect estimates [*Q* statistic (2 degrees of freedom), 0.236; *p* = 0.902].

## Discussion

From public and occupational health perspectives, it is important to address whether the inclusion of plasticizers in household goods and their occurrence in common environments increase risk of asthma and allergies to the public and to certain employees. There are two relevant scientific questions: *a*) Can phthalates, particularly DEHP and its metabolites, cause immunologic effects leading to an increased risk of asthma and allergies? *b*) Are phathalate levels encountered in common and occupational environments sufficient for increasing the risk of asthma and allergies?

### Validity of results

We selected the studies using a clearly defined search strategy. We also used secondary references cited by the articles identified in the primary search. Two observers independently checked the eligibility of the studies according to an *a priori* set of criteria. The small number of studies in the meta-analyses did not allow any formal assessment of potential publication bias; that is, there is a possibility that manuscripts with positive findings were more likely to be published than those with negative findings. Also, some validity issues were common to all epidemiologic studies because of the nature of the study questions. There was a possibility of confounding if some known or unknown determinants of asthma and/or allergies were related to presence of PVC surface materials or phthalates in the environments of interest. All the epidemiologic studies in children adjusted for several potential confounders, as shown in Table 2, but there could also be some residual confounding. Dampness and mold problems in conjunction with PVC materials are a complex issue. There is evidence that dampness enhances both microbial growth and degradation of PVC materials, both of which are potential determinants of asthma. The Swedish case–control study (Bornehag et al. 2005) showed an association between PVC materials and asthma and allergies, mainly in the presence of dampness problems. This could mean that dampness is needed for any relevant emissions from PVC materials and/or for dampness-induced microbial growth and PVC degradation products to interact in causing asthma and allergies. All the epidemiologic studies included in the meta-analyses adjusted for dampness and mold problems (Table 2), and therefore, dampness problems are not likely to confound the observed relationship between PVC and/or phthalates. The role of interaction cannot be properly addressed with the present data.

### Causal inference

**Figure 2:**
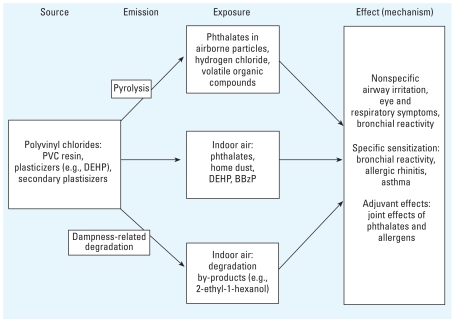
A framework for causal inference illustrating a relevant source–emission–concentration–exposure–effect pathway.

Figure 2 presents a framework for causal inference by illustrating a relevant source–emission–concentration–exposure–effect pathway, which is useful for judging the weight of evidence and need for further research. The first challenge in causal inference is the identification of the specific agent(s). Øie et al. (1997) hypothesized that MEHP, the primary hydrolysis product of DEHP, could serve as a specific causal agent. They proposed that MEHP, the primary hydrolysis product of DEHP, mimics the inducing prostaglandins (PG) PGD(2), 9α,11βPGF2, and PGF2α and thromboxanes in the lungs, thereby increasing the risk of inducing inflammation in the airways, which is a characteristic of asthma.

Laboratory studies have shown that many phthalate compounds administered to mice by subcutaneous injection (Larsen et al. 2001a, 2001b, 2002) or by inhalation (Hansen et al. 2007) exert an adjuvant effect on the immune response to exposure to a coallergen. The extent of increased response appeared to be associated with the length of the carbon chain (Larsen et al. 2001a). The inhalation studies mimicked the human route of exposure to airborne phthalates and provided a measure of NOEL (0.03 mg MEHP/m^3^) (Hansen et al. 2007). This NOEL was calculated to exceed levels encountered in common environments as described by Larsen et al. (2004) and Hansen et al. (2007), and also by Larsen et al. (2007), which was published since the cutoff date for our literature search and thus was not included in the systematic review.

Ideally, we should have knowledge of sources and emission rates of phthalates in real-life conditions, concentration measurements preferably at human breathing zones, and biomarkers of exposure. Also required are exposure–effect relation studies, which use appropriate measures of exposures and defined outcomes, such as an immunologic parameter or clinical sign. Present knowledge, though sparse, is summarized below, highlighting areas that require investigation to enable a rational approach to further research.

### Sources

Although the main source of exposure for DEHP and other phthalates in the general population is diet (Clark et al. 2003; Meek et al. 1994; Peterson et al. 2000; Wormuth et al. 2006), PVC materials in the environment constitute an important source for inhalational exposure. Several million tons of phthalates are used each year worldwide in the production of PVC and other plastics. For example, the use of DEHP in Europe in 1997 is estimated at 476,000 tons, of which about 97% is used as plasticizer in polymers, mainly PVC (European Chemicals Bureau 2006), for use in outdoor products (about 22%) or indoor products (462,000 tons). The typical concentration of DEHP in plasticized PVC is 30% (RAR 2006). Thus, sources of the phthalates hypothesized to cause asthma and allergies exist in our common and occupational environments.

### Emission

Little is known about the emission rates of phthalates from interior surface materials in normal indoor environmental conditions. Øie et al. (1997) found that phthalates, including DEHP, migrate from PVC tiles to house dust, and inhalation of particles containing phthalates is a plausible route of exposure, especially among children. Heating or burning PVC materials releases phthalates and other combustion products into indoor and ambient air, as shown in studies on meat wrapper’s asthma. There is evidence that dampness enhances degradation of PVC flooring, resulting in indoor air concentrations of 2–32 μg/m^3^ of 2-ethyl-1-hexanol, a hydrolysis product of DEHP (Norbäck et al. 2000; Tuomainen et al. 2004). Pyrolysis and dampness-related degradation of PVC materials also cause emission of various other chemicals that may influence airway irritation and inflammation and increase risk of asthma and allergies, but unfortunately, the available studies were unable to separate these effects from those of phthalates.

### Concentrations

Only a few studies examined indoor air and home dust concentrations of phthalates. Øie et al. (1997) reported that DEHP is the predominant phthalate species in both total suspended dust (mean, 64 μg/ 100 mg; range, 10–161 μg/100 mg) and organic fraction (82 μg/100 mg; range, 11–210 μg/100 mg) in homes of children 0–2 years of age. In the 38 samples of sedimented dust, DEHP accounted for 32–97% (mean, 69%) of the total amounts of phthalates in total dust. Øie et al. (1997) inferred that inhalation of aerosols of DEHP adsorbed to particulate matter is as important as, or more important than, vapor-phase exposure. As part of the case–control study, Bornehag et al. (2004b) reported concentrations of six phthalates in house dust. The median concentration of DEHP was 0.770 mg/g dust (mean, 1.310 mg/g). Adibi et al. (2003) measured levels of phthalates in 48-hr personal air samples collected from homes of pregnant women in New York, New York, and Krakow, Poland. The range of DEHP was 0.05–0.41 μg/m^3^ (mean ± SD, 0.22 ± 0.10 μg/m^3^) in New York and 0.08–1.1 μg/m^3^ (0.43 ± 0.24 μg/m^3^) in Krakow. Nielsen et al. (1989) reported DEHP concentrations in different work sites in the polyvinyl processing industry ranging from 20 to 2,000 μg/m^3^.

### Exposure

Inhalation exposure of phthalates may be assessed directly from air in the breathing zone, or indirectly from house dust or by analysis of urinary biomarkers, but information on indoor air concentrations of phthalates is limited. Wormuth et al. (2006) presented scenario-based estimates of overall exposure to eight phthalates in the general population. Exposure to DEHP is mainly from the diet, particularly fatty foods (e.g., dairy, fish, oils), from consumer products and medical procedures, and to a minor extent, by inhalation, via indoor air and household dust. In occupational settings, the inhalation of phthalates via fumes from heated PVC most likely constitutes a comparatively larger fraction of exposure for workers.

### Effects

Laboratory experiments in mice have demonstrated that phthalate compounds exert a modulatory effect on the immune response to exposure to a coallergen (Hansen et al. 2007; Larsen et al. 2001a, 2001b, 2002b, 2003).

There is evidence that occupational exposures to pyrolysis products of phthalate and other combustion products increase the risk of asthma. Case reports identified and verified cases of asthma that were very likely caused by fumes from hot-wire cutting of PVC film or in combination with fumes from thermo-activated price labels [Appendix Table A; available online in Supplemental Material (http://www.ehponline.org/members/2008/10846/suppl.pdf)]. These reports also provide evidence that strong work-related respiratory symptoms may be experienced without specific airway reactivity to these fumes.

Results of the epidemiologic studies in adults (Table 1) are consistent with case reports on the effects of PVC film fumes on asthma and respiratory symptoms. Furthermore, epidemiologic studies in adults provide some evidence of a relation between interior PVC surface materials and the risk of asthma. Findings on effects on lung function are contradictory; in a study of 240 adult Third National Health and Nutrition Examination Survey participants, Hoppin et al. (2004) showed an association between urinary mono-butyl phthalate levels and decrements of FVC, FEV_1_, and peak expiratory flow measurements, and between monoethyl phthalate levels and decrements of FVC and FEV_1_, among men but not among women. MEHP levels were not associated with any of the pulmonary function parameters.

There is suggestive evidence of the role of dampness as an inducer of PVC degradation and emissions of 2-ethyl-1-hexano, although no bronchial reactivity to PVC was shown by a single human experiment (Tuomainen et al. 2006). The epidemiologic studies in children show an association between presence of PVC surface materials in the home and risk of asthma and allergies (Table 2). A recent case–control study among Bulgarian children provides further evidence for a relation between DEHP concentration in house dust and the risk of wheezing, rhinitis, and/or asthma (Kolarik et al. 2007).

### Summary and future research

Some evidence supports the hypothesis that phthalate emissions from PVC materials increase the risk of asthma and allergies. High levels of phthalates from PVC products can modulate the murine immune response to a coallergen. Heated PVC fumes possibly contribute to development of asthma in adults. Epidemiologic studies in children show associations between indicators of phthalate exposure in the home and risk of asthma and allergies.

This putative association between PVC and risk to health is potentially very important from both public and occupational health perspectives. However, we need greater understanding of the factors that may cause PVC materials to induce human adverse effects.

Research is required in several areas to gain insight into the mechanisms of emission, exposure, and toxicity of the chemical species released from PVC materials. In brief, it would be beneficial to improve knowledge of the following:

- Emission rates and migration of phthalates from different types of PVC surface materials. This would enable those with high emission rates to be eliminated from the market.
- The influence of dampness and temperature on degradation of PVC materials. Product design may enhance the effects of dampness on PVC materials, or the presence of PVC materials may enhance microbial growth in indoor surfaces, either of which may influence human exposure.
- Investigation of relationship of doses used in animal experiments with human exposures. Recent inhalation studies in mice have correlated experimental exposures with estimated human exposures (Hansen et al. 2007; Larsen et al. 2004, 2007). Their conclusion was that nonoccupational human exposures to DEHP are unlikely to cause an adjuvant effect or allergic inflammation in the lung. Additional mechanistic studies, in association with human exposures, are required to support these findings.
- Migration of phthalates into house dust and resuspension of phthalate-containing particles into breathing zone.
- Monitoring effects of methods to reduce indoor exposure, for example, by vacuum cleaning and by increasing air change, and use of fume protective masks in occupations.
- Immunomodulatory effects of phthalates in humans. Effects of short-term inhalation exposure to phthalates from PVC materials could be studied in controlled chamber studies, as demonstrated by Tuomainen et al. (2006). Such studies could be used to develop biomarkers of exposure and early immunologic effects for use in epidemiologic studies.
- The effects of exposure to phthalates in homes and work places on the risk of asthma and allergies, both in children and in adults, by conducting large-scale epidemiologic studies, preferably population-based cohort and incident case–control studies in different populations and housing conditions.
- Genetic susceptibility to the effects of phthalates on asthma and allergies, by integrating assessments of gene–environment interactions in epidemiologic studies.

## Figures and Tables

**Table 1:** Summary of the 10 epidemiologic studies on the relationships between exposure to phthalates and PVC materials and the risk of asthma, allergy, and related respiratory outcomes in adults, Medline search from 1950 through May 2007.

Reference, location	Study design	Study population	Exposure	Outcomes	Results	Comment
Polakoff et al. (1975), USA	Cross-sectional study	17 meat wrappers: 21 office personnel and store clerks as a reference group	Inhalation exposure to pyrolysis products of PVC film; assessment based on job category (meat wrappers exposed) and questionnaire information	Symptoms, signs based on questionnaire information; pre- and postshift spirometry: FVC, FEV_1_, PEF, FEF_25_, FEF_50_, FEF_75_, FEF_90_	Exposed had a higher prevalence of cough ever (47.1% vs. 23.8%), work-related shortness of breath (23.5% vs. 0%), wheezing (5.9% vs. 0%), eye watering and itching (17.6% vs. 9.5%), nasal and pharyngeal symptoms (29.4% vs. 4.8%), allergies (11.8% vs. 9.5%), and decline over shift in FEV_1_ (*p* < 0.05) and FEF_50_ (*p* < 0.05)	Frequency matching of reference group but no adjustment for potential confounders
Falk and Portnoy (1976), Houston, TX, USA	Cross-sectional study	145 meat wrappers; 150 checkers and 150 meat cutters as a reference group	Inhalation exposure to pyrolysis products of PVC film; assessment based on job category and interview information	Symptoms, signs based on questionnaire information	Symptom prevalences in exposed vs. checkers and cutters: shortness of breath (16% vs. 4% and 4%; *p* < 0.05), wheezing (12% vs. 5% and 7%; NS), chest pain (17% vs. 5% and 7%; *p* < 0.05), bronchitis (31% vs. 19% and 13% *p* < 0.01), pneumonia (36% vs. 27% and 9%; NS), and pleurisy (33% vs. 16% and 9%; *p* < 0.01)	Frequency matching of reference group but no adjustment for potential confounders
Andrasch et al. (1976), Portland, OR, USA	Cross-sectional study	96 meat wrappers	Inhalation exposure to pyrolysis products of PVC film; assessment based on job title (meat wrappers exposed) and questionnaire information	Symptoms and signs based on questionnaire information (response rate, 58%); and on bronchial provocation test to PVC fumes and price-label adhesive fumes for 14 workers	69% had work-related respiratory, mucosal, or system symptoms; 3 of 11 workers developed a mean decrease of 25% in FEV_1_ after exposure to PVC fumes; 9 of 13 workers developed a 49% decrease in FEV_1_ and 40% decrease in FVC after exposure to price-label adhesive fumes	77% of symptomatic workers reported improvement on weekends and during vacations; no adjustment for potential confounders
Brooks and Vandervort (1977), Ohio, USA	Cross-sectional study	44 workers in retail food industry: 24 exposed meat wrappers; 20 office workers and store clerks as a reference group	Inhalation exposure to pyrolysis products of PVC film and thermoactivated price-label adhesive fumes	Symptoms and signs based on questionnaire information, spirometry (FVC, FEV_1_, MMF, VC_50_, and VC_25_)	Exposed vs. reference: cough, 37% vs. 10%; dyspnea, 29% vs. 10%; wheezing, 12% vs. 0%; asthma/ allergy, 17% vs. 5%; nasal symptoms, 14% vs. 0%; no differences between pre- and postshift lung function tests	Exposed attributed symptoms to PVC film fumes rather than price-label adhesive fumes; no adjustment for potential confounders
Eisen et al. (1985), Boston, MA, USA	Cohort study	83 workers in the retail food industry: 40 exposed to hot-wire or cool-rod fumes, and 43 as a reference group	Inhalation exposure to pyrolysis products of PVC film; assessment based on job title: meat wrappers, meat cutters, and delicatessen product workers exposed	Change in FEV_1_ over time (mL/year)	No difference in FEV_1_ change between the exposed and reference group; interaction term “hot-wire exposure* asthma/allergy,” 76 mL/year, *p* < 0.06	Workers with asthma or allergy may be more susceptible; adjusted for age, smoking, and asthma/allergy
Markowitz (1989), Plainfield, NJ, USA	Cohort study	86 firefighters: 66 exposed, 20 as a reference group	Exposed to burning PVC at a warehouse fire	Occurrence and severity of respiratory symptoms based on questionnaire information: cough, wheeze, shortness of breath, and chest pains 5–6 weeks and 22 months after exposure	Exposed scored significantly higher for all symptoms after 5–6 weeks and all except wheeze after 22 months	No adjustment for potential confounders
Nielsen et al. (1989), Denmark	Cross-sectional study	39 workers in a PVC processing plant: 20 exposed employed as machine attendants and calendar operators, 19 unexposed	Exposed to PVC thermal degradation products and phthalic acid esters	Symptoms, signs based on questionnaire information, bronchial provocation test, specific serum IgGs and IgEs, spirometry (VC, FEV_1_, FEF_50_, FEF_75_)	Exposed vs. reference: conjunctivitis, 25% vs. 0% (*p* < 0.02); rhinitis, 20% vs. 10%; unspecific bronchial hyperreactivity, 25% vs. 5%; dry cough, 45% vs. 0% (*p* < 0.001); asthma, 10% vs. 0%; one positive reaction in bronchial provocation; one exposed had IgG against phthalic anhydride; no differences in lung function parameters	Adjustment for age, height, and smoking habits
Norbäck et al. (2000), Sweden	Cross-sectional study	87 workers in four hospitals: 50 residing in exposed buildings and 37 residing in reference buildings	Two exposed buildings with signs of dampness-related degradation of DEHP in PVC flooring and presence of 2-ethyl-1-hexanol in indoor air; two reference buildings	Doctor-administered questionnaire on presence of asthma symptoms, wheezing, and/or attacks of breathlessness	Exposed (yes/no): asthma symptoms, AOR, 8.6 (95% CI, 1.3–56.7)	Adjusted for sex, age, atopy, current smoking, building dampness at home and at work
Tuomainen et al. (2004), Finland	Repeated cross- sectional study before and after intervention	Office building with 148 workers: first survey, 92 participants; second survey, 115 participants	Before intervention: damp and damaged PVC flooring, 1–3 μg 2-ethyl-1-hexanol per cubic meter of air	Questionnaire information on symptoms and perceived air quality	Index office vs. national rates: eight new cases of asthma in 4 years, 9.2 times more than expected	Intervention included removal of floor coverings, adhesives, and smoothing layers
Jaakkola et al. (2006), southern Finland	Population-based incident case– control study	521 new cases of asthma (21–63 years of age), and 932 population controls	Questionnaire information on presence of plastic wall paper and flooring in the home	Standardized clinical diagnosis of asthma based on history, bronchial challenge, and PEF monitoring	Asthma AOR (95% CI): plastic wall materials at work, < 50% surface vs. none, 1.26 (0.49–3.22); ≥ 50% surface vs. none, 2.43 (1.03–5.75); PVC flooring at work, 1.13 (0.84–1.51)	Adjusted for sex, age, education, smoking, ETS, other surface materials at home and at work

Abbreviations: AOR, adjusted OR; ETS, environmental tobacco smoke; FEF_50_, forced expiratory flow at 50% of vital capacity; FEF_75_, forced expiratory flow at 75%; FEF_90_, forced expiratory flow at 90%; FEV_1_, forced expiratory flow in 1 sec; FVC, forced vital capacity; MMF, maximal midexpiratory flow; NS, not significant; PEF, peak expiratory flow; VC, vital capacity; VC_50_, 50% vital capacity; V_25_, 25% vital capacity.

**Table 2:** Summary of the five epidemiologic studies (in seven articles) on the relations between exposure to phthalates and PVC materials and the risk of asthma, allergy, and related respiratory outcomes in children: Medline search from 1950 through May 2007.

Reference, location	Study design	Study population	Exposure	Outcomes	Results	Comment
Jaakkola et al. (1999), Oslo, Norway	Cohort-based matched case– control study	Children 0–2 years of age: 251 cases of bronchial obstruction and 251 one-to-one matched controls	Blinded investigator assessment: presence of PVC flooring and a quantitative PVC index (range, 0–8)	Case defined as two or more episodes with symptoms and signs of bronchial obstruction or one episode lasting > 1 month	AOR (95% CI): PVC flooring, yes/no, 1.89 (1.14–3.14); PVC index, Q3 vs. Q2 & Q1, 1.34 (0.78–2.30); PVC index, Q4 vs. Q2 & Q1, AOR, 2.71 (1.50–4.91)	Adjustment for other surface materials, sex, parental atopy, having siblings, daycare attendance, breast-feeding, exposure to ETS, dampness problems, maternal education, family income
Øie et al. (1999), Oslo, Norway	Cohort-based case–control study	Children 0–2 years of age: 172 cases of bronchial obstruction and 172 one-to-one matched controls	Air change measurements; low air change rate of < 0.5/hr	Case defined as two or more episodes with symptoms and signs of bronchial obstruction or one episode lasting > 1 month	AOR (95% CI), PVC index > 75th percentile: low air change, 12.6 (1.00–159); high air change, 2.6 (1.02–6.58)	Adjustment for sex, parental atopy, having siblings, daycare attendance, breast-feeding, exposure to ETS, dampness problems
Jaakkola et al. (2000), Espoo, Finland	Population-based cross-sectional study	2,568 children 1–7 years of age	Questionnaire information on presence of plastic wall or flooring material in the home	Questionnaire information on the presence of asthma, allergic rhinitis, respiratory symptoms, infections	AOR (95% CI), plastic wall material (yes/no): asthma, 1.52 (0.35–6.71); rhinitis, 1.20 (0.36–3.97); wheeze, 3.42 (1.13–10.4); cough, 2.41 (1.04–5.63); phlegm, 2.76 (1.03–7.41); nasal congestion, 0.95 (0.33–2.71); nasal excretion, 0.90 (0.32–2.57)	Adjusted for sex, age, highest parental education, single guardian, daycare center attendance, pets, ETS, dampness problems
Bornehag et al. (2004a, 2004b), Varmland, Sweden	Population-based cross-sectional study	10,851 children 1–6 years of age	Questionnaire information on presence of PVC, dampness, and mold problems	Questionnaire information on doctor-diagnosed asthma, rhinitis and respiratory symptoms	AOR (95% CI) for PVC flooring (yes/ no): asthma, 0.98 (0.77–1.24); rhinitis, 1.09 (0.91–1.30) For water leakage (yes/no), asthma,1.23 (0.96–1.58); rhinitis, 1.35 (1.12–1.62) For PVC and leakage (yes/no), asthma, 1.48 (1.11–1.98); rhinitis, 1.22 (0.96–1.55)	Evidence of an interaction between PVC flooring and water leakage on asthma; adjusted for sex, age, allergic symptoms in family, smoking in household
Bornehag et al. (2004b), Varmland, Sweden	Population-based prevalent case– control study	198 cases of persistent asthma, rhinitis, or eczema and 202 popu lation controls; 106 asthma and 79 rhinitis cases and 177 controls	Trained investigator assessment of PVC flooring and bedroom dust concentrations of DEHP, BBzP, and four other phthalates	Baseline and 2-year follow-up surveys; medical examination and case verification	Q4 vs. Q1, AOR (95% CI) BBzP concentration for asthma, 1.87(0.92–3.81); rhinitis, 3.04 (1.34–6.89); eczema, 2.56 (1.24–5.32); DEHP for asthma: AOR (95% CI), 2.93 (1.36–6.34); rhinitis, COR,1.55 (0.73–3.28); eczema, COR, 1.50 (0.76–2.96)	Adjusted for sex, age, smoking at home, type of building, construction period, flooding
Jaakkola et al. (2004), nine cities, Russia	Cross-sectional study	5,951 children 8–12 years of age	Questionnaire information on recent installation of surface materials and furniture	Questionnaire information on current asthma, current wheezing, any allergy	AOR (95% CI), “linoleum”/ PVC flooring (yes/no): past 12 months for asthma, 1.13 (0.44– 2.04); wheeze, 1.36 (1.00–1.86); allergy, 1.31 (1.05–1.65) Earlier for asthma, 1.39 (0.67–2.77); wheeze, 1.21 (0.99–1.59); allergy, 1.22 (1.04–1.45)	Adjustment for age, sex, preterm birth, low birth weight, parental atopy, maternal smoking in pregnancy, exposure to ETS, mother’s and father’s education

Abbreviations: AOR, adjusted OR; COR, corrected OR; ETS, environmental tobacco smoke; Q, quartile.
